# Raman Spectroscopy and X-Ray Diffraction Investigations of Phase Composition of Tiglit Meteorite

**DOI:** 10.3390/ma19030624

**Published:** 2026-02-06

**Authors:** Anna Karczemska, Mariusz Dudek, Bartłomiej Januszewicz, Tomasz Jakubowski, Stanisław Mitura

**Affiliations:** 1 Institute of Turbomachinery, Lodz University of Technology, Wolczanska 217/221, 93-005 Lodz, Poland; 2 Institute of Materials Science, Lodz University of Technology, Stefanowskiego 1/15, 90-537 Lodz, Poland; mariusz.dudek@p.lodz.pl (M.D.); bartlomiej.januszewicz@p.lodz.pl (B.J.); 3 Polish Meteorite Society, Bedzinska 60, 41-200 Sosnowiec, Poland; illaenus@gmail.com (T.J.)

**Keywords:** meteorite, aubrite, Raman spectroscopy, SEM EDS, XRD

## Abstract

Tiglit is an aubrite meteorite which fell in 2021 in Morocco. Fragments of the Tiglit meteorite, recovered shortly after its fall, were analyzed for phase and chemical composition using scanning electron microscopy with energy-dispersive X-ray spectroscopy, Raman spectroscopy, and X-ray diffraction. These studies confirmed the presence of pyroxene (enstatite), olivine, plagioclase, sulfides, carbon phases (seldom reported in aubrites), and iron oxides. Unexpectedly, calcite and polymorphic SiO_2_ phases were also detected. The formation of calcite is related to the terrestrial alteration of oldhamite—a sulfide present in aubrites—after the fall.

## 1. Introduction

The Tiglit aubrite [1,2] fell on 10 December 2021, in southern Morocco, approximately 70 km west of the city of Tan-Tan. The fall was accompanied by a bright fireball and audible explosions at around 8:30 PM local time. The first fragments were recovered by local meteorite hunters days after the event (MetBull 111). Due to the fragile nature of aubrites, most specimens were fragmented. Some pieces were covered by an unusual, translucent, and glossy fusion crust, which exhibited a bubbly texture and a yellow-to-brown coloration. Observation of the fall and recovery of material only a few days after [1] provided the opportunity to study exceptionally fresh material of an aubrite for cosmochemical research. This enables a detailed investigation of volatile elements and fragile mineral phases typically altered in older finds. Its official classification was confirmed by the Meteoritical Society and published in the Meteoritical Bulletin in 2023.

Aubrites are a rare group of achondrites [3]. To date, there have been only 12 observed aubrite falls and 84 finds, predominantly from hot and cold deserts. The group is named after the first observed fall in Aubres, France, in 1836. Their intrinsic fragility, often unusual appearance, and the frequent absence of a typical black fusion crust make them particularly difficult to recognize and recover in the field.

Also known as enstatite achondrites, aubrites are primarily composed of nearly pure, magnesium-rich orthopyroxene (enstatite—Mg_2_Si_2_O_6_), which constitutes 75% to 98% of the bulk rock composition. Minor silicate phases include forsterite (Mg_2_SiO_4_), diopside (MgCaSi_2_O_6_), and albitic plagioclase (NaAlSi_3_O_8_). Accessory phases include nickel–iron alloys (kamacite—α-(Fe,Ni) and taenite—γ-(Ni,Fe)) and a diverse suite of sulfides such as troilite (FeS), alabandite (MnS), daubréelite (Fe^2+^Cr^3+^_2_S_4_), niningerite (MgS), and oldhamite ((Ca,Mg)S). The presence of minerals like oldhamite and the overall mineral assemblage indicates that aubrites are formed in a highly reducing environment, distinct from that of most other meteorite groups.

Texturally, aubrites are coarse-grained rocks with a characteristically white interior due to the abundance of enstatite, often containing visible sulfide and metal inclusions. They exhibit two main textures, unbrecciated (e.g., Shallowater) and brecciated, with the latter being predominant among known samples. Brecciated aubrites are typically classified as fragmental or regolith breccias. The shock stage of most aubrites ranges from S3 to S4 on the Stöffler scale [4,5].

Aubrites crystallized very early in the history of the Solar System; their age is close to 4.56 Ga, as documented for samples such as Shallowater, Peña Blanca Spring, Norton County, and Bishopville [6,7,8]. Although their crystallization ages are broadly similar, aubrites show highly variable cosmic ray exposure ages, indicating multiple and temporally distinct ejection events from their parent body (likely 4 to 9 impact-induced break-up events) [5].

For example, Shallowater has a short exposure age of ~20–30 Ma, whereas Norton County and Mayo Belwa display much longer exposure histories, exceeding 100 Ma, reflecting their prolonged residence as meteoroids in interplanetary space. The cosmic-ray exposure ages of different samples are extensive, ranging from 10 to 120 Ma, which is among the longest recorded for achondrites. The origin of the aubrite parent body remains a subject of debate, though they have traditionally been linked to enstatite chondrites as potential precursor materials.

Meteorites represent a unique class of natural composite materials, forged under extreme thermo-mechanical conditions not replicable in terrestrial laboratories. A comprehensive characterization of their complex, multi-scale microstructures is therefore fundamental to understanding their origin and evolution. The intricate interplay between mineralogical phases and their chemical compositions serves as a robust record of the parent body’s formation and subsequent alteration history. Therefore, the present study used a correlative, multi-analytical materials science approach combining X-ray diffraction (XRD), scanning electron microscopy with energy-dispersive X-ray spectroscopy (SEM-EDS), and Raman spectroscopy to comprehensively investigate the chemical and phase composition of the Tiglit meteorite. The results of these measurements will allow for the interpretation of the sequence of cosmochemical and physical processes occurring in the volume of the meteorite, thus providing a new perspective on the processes occurring in the material in extraterrestrial conditions. This specimen was selected because the Tiglit meteorite, despite its classification, has not yet been subjected to such a comprehensive and targeted investigation (as, for instance, the meteorite Ribbeck, which fell in Germany, close to Berlin, in January 2024). The present study aims to fill this knowledge gap by providing fundamental data on the composition and microstructure of this unique extraterrestrial material.

Based on previous studies, it is apparent that the Ribbeck and Tiglit meteorites share a broadly similar mineralogical framework—both dominated by Fe-free enstatite, forsterite, troilite, various sulfides, and amorphous carbon phases—with the authors also highlighting that they also exhibit notable differences. Ribbeck contains plagioclase, maskelynite, rutile, and a wider range of sulfides, whereas Tiglit includes clinopyroxene (diopside), Fe oxides, and magnetite and shows a higher abundance of carbonaceous material [2]. The authors indicate that no plagioclase, feldspar, or silica polymorphs have been detected in Tiglit.

Kołodziej et al. [9] describe that Raman spectra show that Ribbeck mainly contains Fe-free enstatite (orthopyroxene), Fe-free forsterite, and Na-rich feldspar (albite). EDS results indicate that these three main phases are accompanied by sulfides, most of which do not occur on Earth. The sulfides include troilite, brezinanite (daubreelite/zolenskyite), alabandite, oldhamite (CaS), and phases of the wassonite/heideite group. Metallic phases are also present. Si-rich kamacite occurs next to sulfides. Schreibersite that encloses kamacite and troilite crystals is observed. These minerals indicate a highly reducing environment. Previous authors have also suggested that Cr–Fe alloys could have formed during atmospheric entry melting, which also caused the sulfur to evaporate. Eyewitnesses who searched for Ribbeck reported a noticeable sulfur smell near fresh fragments. Bischoff et al. [10] report that the dominant phase is nearly-FeO-free enstatite, with a significant abundance of albitic plagioclase, minor forsterite, traces of FeO-free diopside, K feldspar, and a S-bearing, K feldspar-like phase. Ribbeck also contains metals, schreibersite, and sulfides (or their alteration products). Among the sulfides, troilite is abundant. Reported phases also include djerfisherite, alabandite, oldhamite, and portlandite. Only a few grains of oldhamite are present, and most of them show weathering with latticelike textures. One likely alteration product is portlandite [Ca(OH)_2_]. Among the opaque components, two phases appear to have been altered in a wet environment. One of these phases could be cronusite (Ca_0.2_CrS_2_xH_2_O). It may form from caswellsilverite (NaCrS_2_). Daubreelite (FeCr_2_S_4_) or brezinanite (Cr_3_S_4_) cannot be ruled out as possible precursors before alteration. The other altered phase may have been a Ti-rich sulfide before short-term terrestrial, aqueous alteration that lasted only about six days (the fall occurred onto snow that then melted).

In the broader planetary context, aubrites may come from proto Mercury, whose mantle was blasted off by a giant impact in the early Solar System [9]; aubrites would then be small fragments of that ancient mantle. In the last five years, three aubrites have fallen worldwide: Tiglit (2021), Rantila (2022), and Ribbeck (2024). This occurred about fifty years after the previous aubrite fall at Mayo Belwa (1974).

The present work also serves as a direct continuation of the authors’ recent investigation into the Ribbeck aubrite [11], thereby enabling a unique comparative analysis of two pristine, recently fallen samples from this rare achondrite group. In that study, an interesting result predicted by Bischoff [10] was the discovery of elemental sulfur identified by Raman spectroscopy; Raman and SEM–EDS were used to characterize the phases present in the sample.

## 2. Materials and Methods

The Tiglit meteorite fragments under study were obtained from a local meteorite hunter in Morocco. They were found within days after the fall and stored in a dry environment. Tiglit fragments with representative textures characteristics of Tiglit petrography were used. The samples were selected based on the presence of potentially interesting features, including macroscopically visible sulfide grains on their surfaces. No macroscopic signs of weathering were observed on the examined fragments.

The chemical and phase composition of the Tiglit meteorite fragments was studied using techniques such as energy dispersive X-ray spectroscopy (EDS), X-ray diffraction (XRD), and Raman spectroscopy. The EDS studies were performed on a JSM-6610LV (JEOL, Tokyo, Japan) microscope integrated with an EDS X-MAX 80 (Oxford Instruments, Abingdon, UK) analyzer. The measurements were carried out at an accelerating voltage of 5 kV. A PANalytical Empyrean diffractometer (Malvern Panalytical Instruments Ltd., Worcestershire, UK) was used to analyze the phase composition. The XRD patterns were recorded using CoKα (1.790307 Å) radiation at 40 kV and 45 mA, at room temperature. The diffraction angle was scanned between 20 and 100 degrees, with a step size of 0.05 degrees. The measurements were carried out for a relatively flat fragment of the Tiglit meteorite surface and for fragments powdered in an agate mortar. To eliminate the possibility of contamination of the powder, the mortar components were washed beforehand using an ultrasonic cleaner for 30 min in an acetone bath, and then again in an ethyl alcohol bath.

The last device used in the research was the Renishaw inVia Raman microscope (Gloucestershire, UK) equipped with a 532 nm laser arranged in a backscattering geometry. The measurements were carried out in air at room te mperature using an objective lens with a magnification of 50×-diameter of a spot size of ~1 μm [12]. The investigated wavenumber ranged from 100 to 3200 cm^−1^. The remaining parameters (laser power in the range from 4 mW to 20 mW, exposure time, and accumulation in the range from 10 s to 100 s) were selected to obtain good-quality spectra without introducing phase changes at the examined point of the meteorite.

## 3. Results

### 3.1. SEM–EDS Measurements

**Figure 1:**
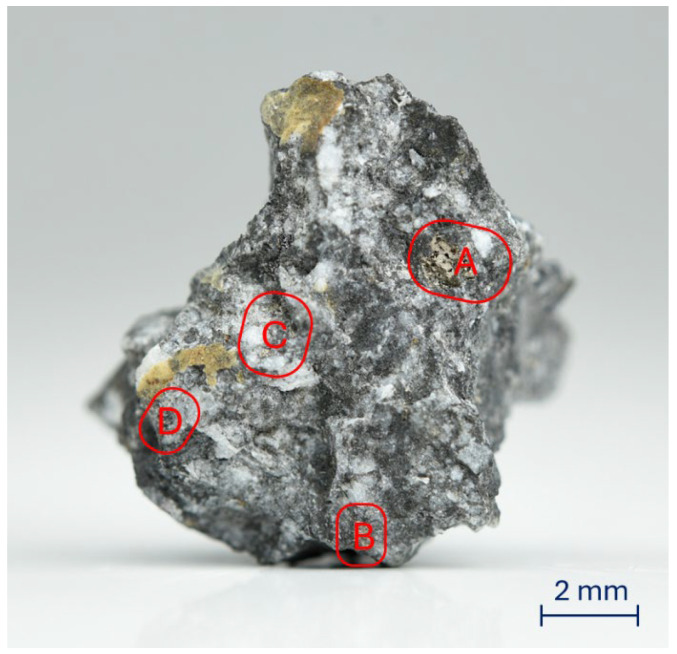
Macroscopic photo of a fragment of the Tiglit meteorite with marked places (A,B,C,D) where sulfides may occur.

The Tiglit meteorite fragment shown in Figure 1 was analyzed for its chemical and phase composition. Initial organoleptic evaluation allowed the isolation of several places on its surface (Figure 1) where sulfides may occur. To confirm these assumptions, an EDS analysis of the chemical composition was performed in these places.

**Figure 2:**
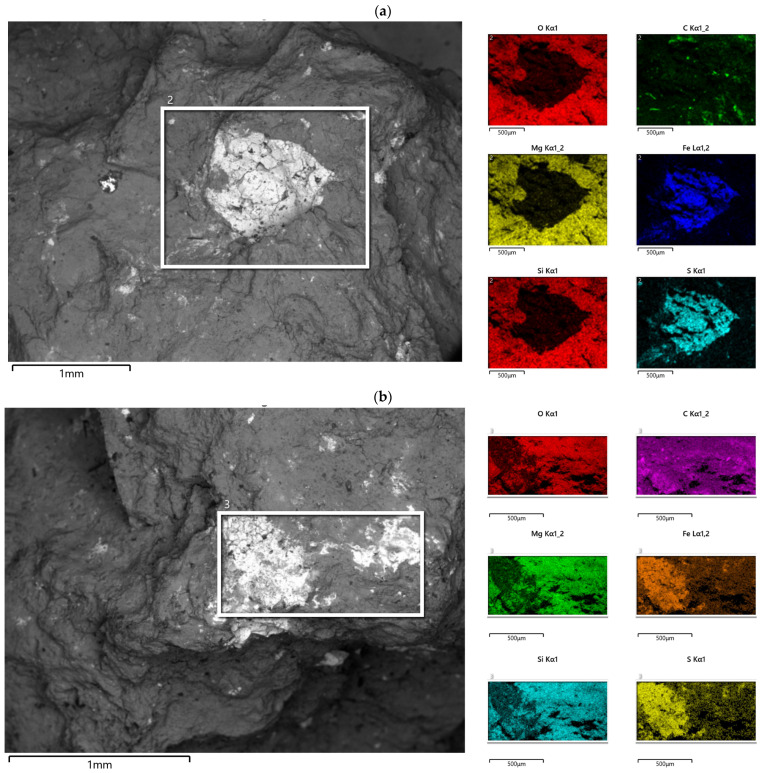
Examples of chemical composition maps made for area A (**a**) and area B (**b**) marked in Figure 1.

Figure 2 shows chemical composition maps obtained using the EDS technique for two exemplary places selected for research (Figure 1). Analysis of these maps confirms the previous assumption about the presence of sulfides in the locations selected for research, indicating that these sulfides may be iron sulfides—for most maps, the presence of sulfur in the locations studied coincides with the map of iron presence, with the simultaneous absence of oxygen, magnesium, and silicon in these locations (Figure 2a). Nevertheless, it is possible to find places where the identified sulfur and iron are accompanied by the presence of oxygen, magnesium, and silicon (Figure 2b).

In order to quantitatively describe the heterogeneity of the chemical composition of the examined fragment of the Tiglit meteorite, point analyses of its chemical composition were performed. As shown in Table 1, in addition to the elements already discussed in the context of maps (Figure 2), such as sulfur, iron, oxygen, magnesium, and silicon, nitrogen, sodium, aluminum, copper, titanium, chlorine, potassium, and calcium can be found in the meteorite. For most of the points or surfaces for which measurements were made, the content of these elements is negligible/negligible, but there are also places where their presence is at the level of 24 at.%, as is the case with titanium in area C.

The results discussed above reveal the chemical composition of the meteorite’s surface layer. EDS analysis was also performed on the powdered meteorite fragment prepared for XRD measurements (results in Section 3.3). This analysis confirmed the presence of chemical elements already known from the meteorite’s surface. However, it is noteworthy that regardless of the portion of meteorite powder examined (Table 1), the calcium level did not drop below 6.5 at.%, while on the surface, calcium was identified at a level of 0.2 at.% only in one spot. These results demonstrate that the examined fragment of the Tiglit meteorite is highly diversified in terms of its chemical composition.

### 3.2. Raman Spectroscopy Measurements

**Figure 3:**
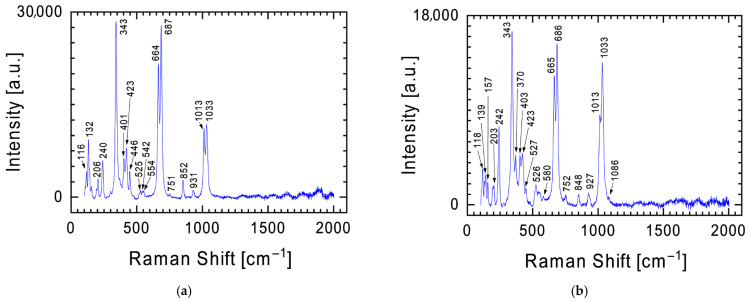
Examples of the two most common Raman spectra obtained during measurements of meteorite surface—Raman spectra (**a**,**b**) characteristic for pyroxenes with characteristic bands at ~240 cm^−1^, ~343 cm^−1^, ~664 and 687 cm^−1^, and ~1013 and 1033 cm^−1^.

The dominant element in the conducted analyses of the chemical composition is oxygen (Table 1). This means that, looking from the perspective of the phase composition of the examined fragment of the Tiglit meteorite, it will be dominated by silicon and metal oxides. This is confirmed by the results of measurements performed using a Raman spectrometer, where the spectra taken are dominated by bands characteristic of these oxides. Figure 3 presents examples of two Raman spectra of the meteorite’s surface obtained during measurements. In each of them, the bands ~240 cm^−1^, ~343 cm^−1^, ~664 and 687 cm^−1^, and ~1013 and 1033 cm^−1^ are at the forefront, which differ in intensity relative to each other depending on the measurement location. These bands are characteristic of pyroxenes with the general formula XY(Si,Al)_2_O_6_, where X represents Ca, Na, Fe(II), or Mg and Y represents ions of smaller size, such as Cr, Al, Mg, Co, and Ti. Pyroxenes crystallize in two main systems: (i) monoclinic, referred to as clinopyroxenes, and (ii) orthorhombic, referred to as orthopyroxenes. Although the Raman spectra of both types are broadly similar, subtle differences in band positions and intensities occur [13,14,15,16,17,18,19]. Clinoenstatite shows characteristic additional bands near 369 and 431 cm^−1^, while the ~236 cm^−1^ orthoenstatite band is shifted to around 243 cm^−1^ [19]. In the case of Tiglit, the presence of strong bands near 1033, 1013 cm^−1^, and 687, 664 cm^−1^ is consistent with Mg-rich pyroxenes, indicating enstatite as the dominant phase of this meteorite. Variations in band intensity across different areas of the sample likely reflect local heterogeneity in chemical composition and the degree of shock metamorphism.

**Figure 4:**
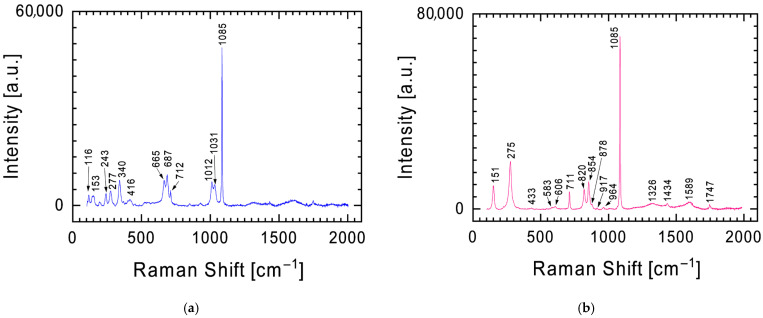
Raman spectrum with the characteristic calcite band at 1085 cm^−1^ against the background of less intense bands for pyroxenes (**a**) and olivine (**b**).

Enstatite is the dominant mineral of the Tiglit meteorite. As shown by the Raman spectra (Figure 3), less intense bands from other minerals can also be observed. In Figure 3b, a band at ~1086 cm^−1^ can be found, which in the spectrum from Figure 4a becomes dominant over the spectrum of pyroxenes. The presence of this band and the second band at 712 cm^−1^ indicates the presence of calcite (CaCO_3_) [12,20,21,22], one of the most common natural carbonate minerals. A powdered meteorite fragment was also examined using a Raman spectrometer (Figure 4b). In addition to the previously mentioned bands, the spectrum revealed two additional bands characteristic of calcite: 151 and 275 cm^−1^. Furthermore, two characteristic Raman lines at 825 and 858 cm^−1^ were identified in the spectrum (Figure 4b), representing fingerprints of olivine with the chemical formula (Mg,Fe)_2_SiO_4_ [23,24,25].

**Figure 5:**
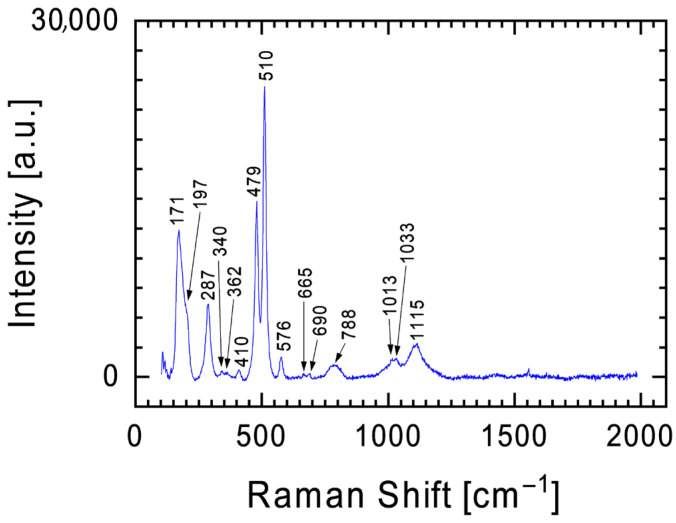
Raman spectrum with characteristic bands at 479 and 510 cm^−1^ for plagioclase.

Another mineral containing silicon and oxygen identified in the Tiglit meteorite is plagioclase, which is a solid solution of two phases: albite (NaAlSi_3_O_8_) and anorthite (CaAl_2_Si_2_O_8_) [16,26,27]. Figure 5 shows a Raman spectrum with characteristic bands at 287, 479, and 510 cm^−1^ for plagioclase [28]. The background to these bands is the bands characteristic of the previously described pyroxenes and calcite. Analysis of the chemical composition results (Table 1) does not allow for a clear determination of which mineral (albite or anorthite) dominates the plagioclase identified in the Tiglit meteorite fragment (sodium and calcium were identified in EDS analysis).

**Figure 6:**
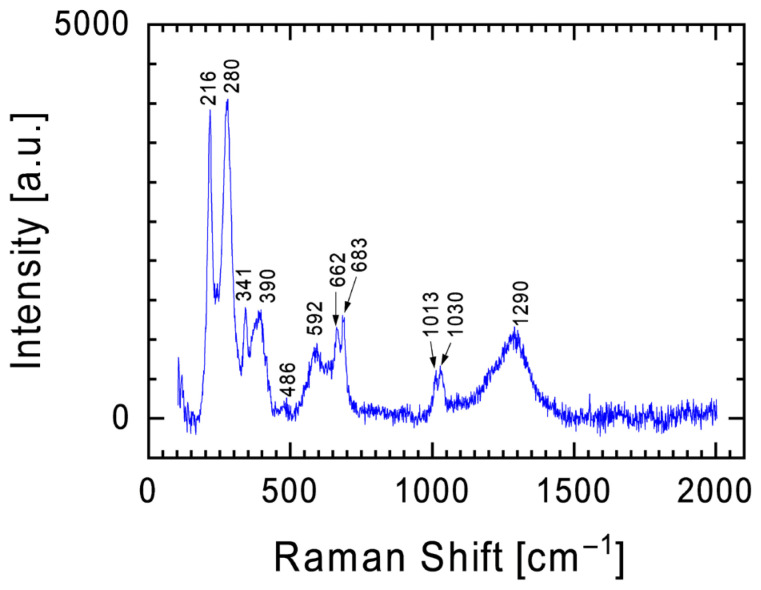
Raman spectrum with characteristic bands for hematite against the background of low-intensity bands characteristic of pyroxenes.

Figure 6 is another example of the dominance of bands characteristic of pyroxenes in the Raman spectra of the Tiglit meteorite. It should be emphasized here, however, that this spectrum is dominated by two bands at 216 and 288 cm^−1^ and a broad band with a maximum at 1290 cm^−1^. These bands are characteristic of hematite [24,25], a common iron oxide compound with the formula Fe_2_O_3_ and widely found in rocks and soils. If it were not for the presence of a broad band with a maximum at 1290 cm^−1^ in the spectrum, analyzing the spectrum from Figure 6 with bands at 216, 278, and 390 cm^−1^, one could hypothesize that these bands may indicate the presence of iron sulfide (FeS) in the meteorite [29].

Further analysis of the spectrum in Figure 6 allows us to distinguish a band at 486 cm^−1^ and 592 cm^−1^. The band at 486 cm^−1^ indicates the presence of microcline (KAlSi_3_O_8_), while the band at 592 cm^−1^ indicates the presence of a calcium–aluminum phase [25,30]. It is worth emphasizing here that the presented Raman spectrum was taken in the D region, for which the point EDS analysis conducted of the chemical composition showed the presence of potassium and calcium.

**Figure 7:**
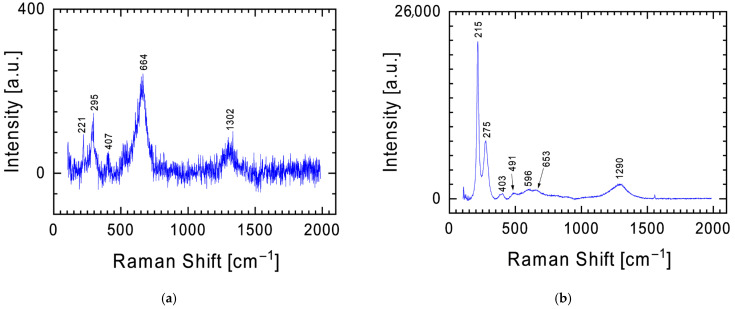
Raman spectra with characteristic bands for iron oxides obtained for the same point on the surface of the examined meteorite fragment at 4 mW (**a**) and 20 mW **(b**) laser power during the measurement.

It should be emphasized here that the presence of bands 216, 288, and 1290 cm^−1^, characteristic of hematite in the spectrum (Figure 7), does not exclude the presence of magnetite (Fe_3_O_4_) in the meteorite (strong band at 664 cm^−1^—Figure 7a) [22]. The phenomenon of magnetite transformation into hematite during measurements using a Raman spectrometer with increasing power of the scattered laser beam is well described in the literature [31,32,33]. While examining the surface of the meteorite fragment, places indicating the presence of magnetite were found (Figure 7a), which, under the influence of increasing the power of the scattered laser beam, were transformed into hematite (Figure 7b). To summarize, the presence of bands characteristic of hematite in the Raman spectrum may indicate its presence in the parent material, the transformation of magnetite as a result of the increase in temperature during the meteor’s entry into the Earth’s atmosphere, or the activation of the sample with too high a power of laser beam used during the measurement.

**Figure 8:**
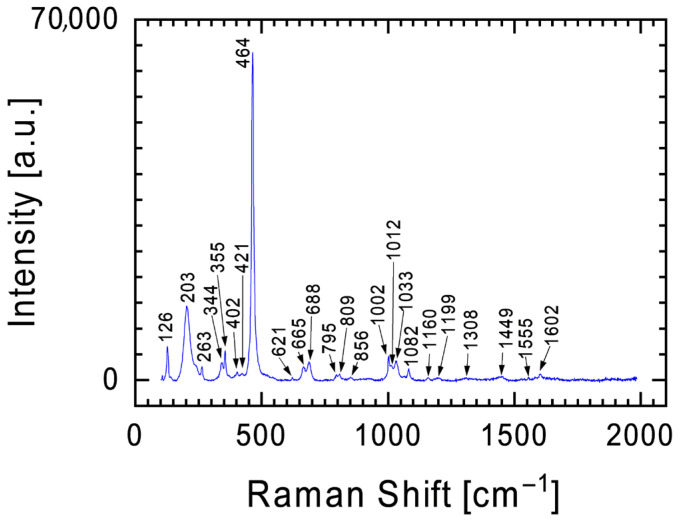
Raman spectrum with characteristic bands at 126, 203 and 464 cm^−1^ which can be attributed to quartz, cristobalite, or trydimite.

An interesting example of the Raman spectrum for the Tiglit meteorite is the spectrum in Figure 8, with three bands at 126, 203, and 464 cm^−1^ coming to the foreground against the background of the previously described bands for pyroxenes. After a detailed analysis of the positions and intensities of the individual bands, these bands can be attributed to silicon oxide without any additives, specifically quartz [34,35]. The Raman spectrum (Figure 8) also includes signals around 203, 355, 402, 620, 795–809, and 1080 cm^−1^, which indicate the presence of high-temperature SiO_2_ polymorphs such as cristobalite and tridymite (this phase is very little active in the Raman spectra, so in the future additional investigations would be important). The coexistence of these phases suggests a dynamic thermal history involving transient heating and rapid cooling, consistent with shock metamorphism. Both SiO_2_ polymorphs are good temperature indicators, being stable in specific conditions. For tridymite, temperature ranges fall between 870 °C and 1470 °C, and for cristobalite, above 1470 °C [36].

In the low-frequency region of the Raman spectra, a number of subtle features appear in which metal sulfide phases may be “hidden”. Bands in this range can correspond to various metal sulfides [29,37] or sodium sulfide [38]. According to the literature, the most intense Raman bands characteristic of metal sulfides typically occur at approximately 160, 185, 215–255, 290, 360, 405, and 480 cm^−1^ [22,29,39,40]. Pyrite, for example, exhibits bands near 340 and 375 cm^−1^, while FeS is characterized by bands at 216, 278, and 390 cm^−1^; other studies, however, report a central Fe sulfide band around 290 cm^−1^ [22,29,41]. In many spectra obtained in this study, multiple bands appear that overlap with these characteristic sulfide positions. In many Raman spectra obtained in the present study, multiple bands are observed that correspond well to the characteristic shifts in sulfide phases. However, considering that the Raman spectra presented and discussed above are rich in bands in this region, it is impossible to clearly distinguish bands characteristic of sulfides. This is especially true since in most literature reports, the bands characteristic of metal sulfides are characterized by low intensity.

**Figure 9:**
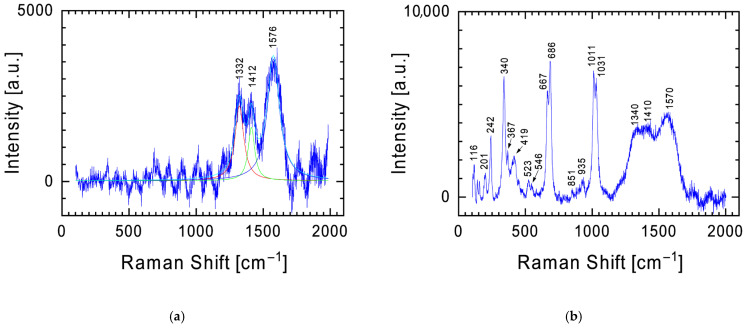
Examples of a Raman spectrum with characteristic bands for carbon forms without (**a**) and against the background of bands characteristic for pyroxenes (**b**). The spectrum from plot (**a**) was deconvoluted using three Lorentz functions.

**Figure 10:**
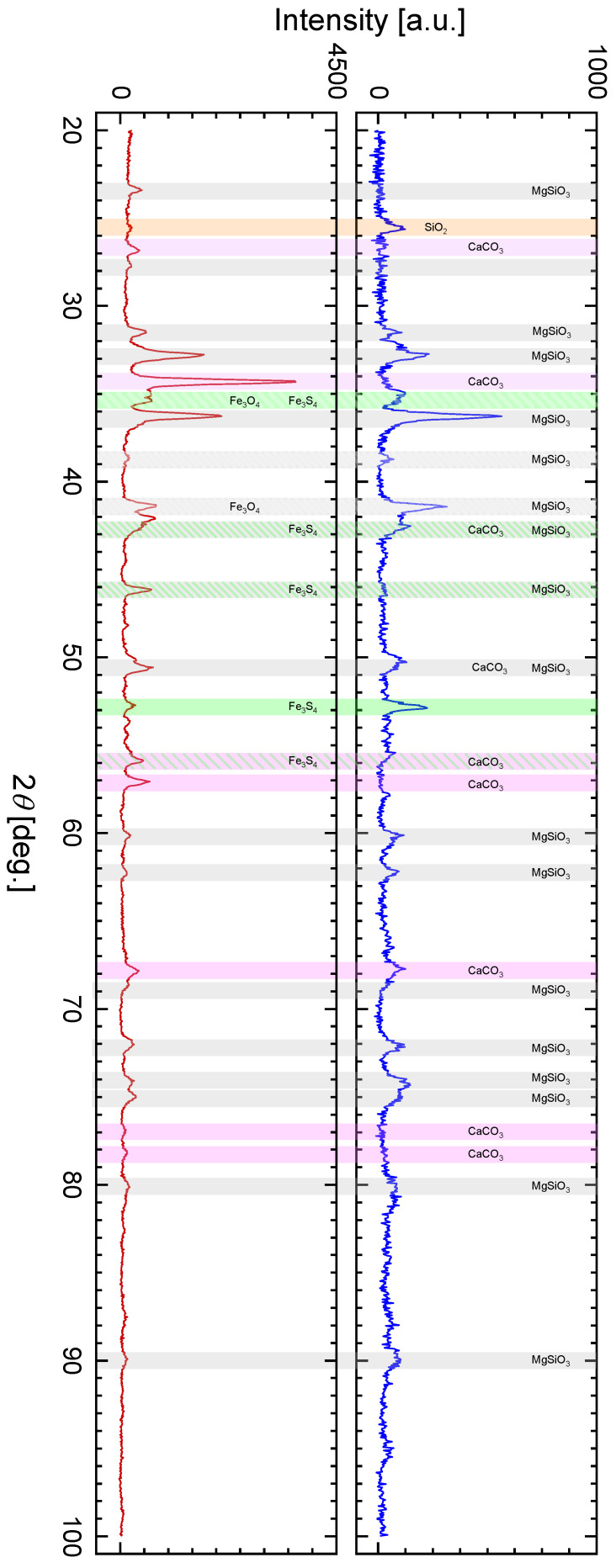
X-ray spectra of Tiglit meteorite obtained from surface (**top**) and powders (**down**).

The presence of characteristic bands for calcite in the Raman spectra of the Tiglit meteorite clearly indicates that the carbon identified in the EDS analysis (Figure 2 and Table 1) occurs, among others, in the form associated with calcium. Considering that the EDS analysis showed a point carbon content of up to 72 at.%, one can expect the presence of carbon in the form of nanodiamonds, nanographite, or disordered carbon [42,43,44]. As shown in the spectrum from Figure 9a, obtained at low laser power, a conglomerate of three bands with maxima at 1332, 1412, and 1576 cm^−1^ is visible, appropriately assigned to nanodiamonds, disordered carbon (in the literature described as the so-called D band), and graphite (the so-called G band). The presence of these bands clearly indicates the presence of ordered carbon species such as nanodiamonds. To eliminate potential doubts arising from visible fluorescence artifacts in the spectrum, measurements were repeated at higher laser powers. After the acquisition of this Raman spectra, bands characteristic of carbon appear against the background of bands characteristic of enstatite (Figure 9b), the dominant mineral in the surface layer of the examined fragment of the Tiglit meteorite. Therefore, the carbon in the meteorite occurs in the ordered form of nanodiamonds. An additional confirmation of this fact may be a weak hump around 2*θ*~51.6 degrees, visible in the XRD spectrum (Figure 10).

### 3.3. XRD Measurements

The final step in determining the phase composition of the Tiglit meteorite was X-ray diffraction analysis. This was performed on the relatively flat surface of the bulk meteorite and its powdered fragment. As shown in Figure 10, they show significant similarity, although due to the lack of perfect flatness of the bulk meteorite fragment, this diffraction pattern exhibits lower peak intensities. The most significant difference between the two diffraction patterns is the absence of a peak at 2*θ* = 34.3° in the diffraction pattern obtained for the bulk meteorite fragment. Comparing the diffraction patterns with the ICDD PDF 4+ database, this peak can be assigned to calcium carbonate (CaCO_3_—PDF Number 01-080-9776). The fact that this peak and the next two peaks for 2*θ* equal to 26.8, 55.8, 57.0, and 67.8° are present in the diffraction pattern of the powdered meteorite confirms earlier observations that in the examined meteorite fragment, the calcium-based structures were enclosed in a “shell” composed of other minerals.

Measurements using a Raman spectrometer indicate that the main component of the “shell” of the examined fragment of the Tiglit meteorite is minerals from the pyroxenes group, but this analysis did not allow for a clear determination of which form of pyroxenes is dominant in it. Analysis of both diffraction patterns presented in Figure 10 shows that the peaks at 2*θ* equal to 23.4, 31.5, 32.7, 36.3, 38.7, 41.4, 42.5, 46.1, 50.6, 60.1, 62.3, 72.0, 74.0, 75.0, and 90.0° should be assigned to enstatite (Mg_2_Si_2_O_6_—PDF Number 00-019-0768) belonging to the group of orthopyroxenes—pyroxenes crystallize within the orthorhombic machine. However, some of these peaks at 2*θ* of 31.5, 32.7, 36.3, 41.4, 42.5, 46.1, and 35.4° can be assigned to clinoenstatite (MgSiO_3_—PDF Number 00-019-0769), a clinopyroxene that crystallizes in the monoclinic crystal system In summary, the presence of both crystalline forms of pyroxenes was confirmed by XRD results.

In addition to the presence of calcium carbonate and pyroxenes, already confirmed, analysis of diffraction patterns allows us to confirm the presence of cristobalite (SiO_2_—PDF Number 00-039-1425) members of the SiO_2_ group—peak at 25.5°—in the examined fragment of the Tiglit meteorite. The peaks at 2*θ* equal to 35.0, 42.5, 46.1, 52.7, and 55.8 may indicate the presence of iron sulfides (Fe_3_S_4_—PDF Number 04-008-7806), while the peaks at 2*θ* equal to 35.0 and 41.4° can be assigned to iron oxides (Fe_3_O_4_—PDF Number 00-019-0629) identified using Raman spectroscopy (Figure 6 and Figure 7).

## 4. Discussion

Orthoenstatite and clinoenstatite can be distinguished by subtle yet diagnostic features in their Raman spectra. Clinoenstatite exhibits additional bands near 369 and 431 cm^−1^, and the orthoenstatite band at 236 cm^−1^ shifts to approximately 243 cm^−1^ [19]. In the spectrum shown, for instance, in Figure 3b, the presence of bands at ~370 cm^−1^ and ~423 cm^−1^, together with a band at ~242 cm^−1^, corresponds to the characteristic Raman signatures of clinoenstatite rather than orthoenstatite. The features at ~370, ~423, and ~242 cm^−1^ suggest contributions from clinoenstatite, but they are not fully diagnostic. Overall, the spectrum is most consistent with a mixture of enstatite polymorphs rather than a purely clino- or orthoenstatite phase, in agreement with the XRD results and with the literature [2].

The identification of calcium carbonate by Raman spectroscopy (Figure 4) and XRD (Figure 10) measurements in the Tiglit aubrite is unexpected because primary carbonates are rare in magmatic achondrites [7]. However, calcite was detected, for instance, in Norton County aubrite as the weathering product of oldhamite [45]. The interpretation is that this phase is terrestrial in origin, formed rapidly after the meteorite’s fall. The mechanism is the ultra-fast weathering of highly unstable aubrite minerals upon exposure to Earth’s atmosphere. Aubrites crystallized under extremely reducing conditions and contain phases such as oldhamite, which react vigorously with atmospheric moisture and CO_2_, producing secondary minerals including calcite [45,46]. This alteration can be initiated within minutes to hours after landing. Therefore, the detected CaCO_3_ should be regarded as a product of rapid terrestrial alteration rather than an indigenous meteoritic constituent.

Given that aubrites are often fragmental or regolith breccias, containing distinct lithic clasts, the identification of unexpected minerals in Tiglit (which is at the moment recognized as the monolithic breccia) might also reflect the incorporation of a foreign clast derived from a more oxidized or volatile-rich parent body. The occurrence of oxide-bearing and FeO-rich clasts in other aubrites [47] demonstrates that aubrite parent bodies (or their collisionally reworked regoliths) could assimilate material with very different redox and petrologic histories. Some authors proposed the hypothesis that, in some aubrites, the material reflects the mixing of components from different parent bodies during collisional events [48,49].

The detection of high-temperature SiO_2_ polymorphs (quartz by Raman spectroscopy measurements (Figure 8) and cristobalite by XRD measurements (Figure 10)) in an aubrite is also particularly unusual. Aubrites are formed under extremely reducing conditions, dominated by nearly-FeO-free enstatite and metal–sulfide phases, where free silica is thermodynamically unstable. Therefore, the presence of high-temperature SiO_2_ polymorphs may reflect post-formational processes, such as shock-induced phase transformation of cristobalite or Si-rich glass, terrestrial oxidation, or metastable crystallization from residual melts. This finding may point to localized redox heterogeneities or late-stage differentiation events in the aubrite parent body, challenging the conventional view that aubritic magmatism excludes free silica phases.

The results of Raman spectroscopy analysis showed that the meteorite contains carbon in a form unbound from calcium (Figure 9). Based on the Raman spectroscopy results, we can conclude that the Tiglit meteorite also contains carbon in the form of nanodiamonds.

These disputed observations underscore that the Tiglit meteorite still holds many secrets. To resolve these uncertainties, additional investigations are essential, including high-resolution microstructural analysis, isotopic studies of the carbonate phase, and comparative research on other aubrites.

## 5. Conclusions

The rapid collection of the Tiglit meteorite shortly after its impact provided a rare opportunity to study material with minimal terrestrial contamination. This is particularly valuable in the context of rare meteorites, where non-destructive or micro-analytical methods are essential to maximize data extraction from limited sample volumes. The meteorite was investigated using complementary materials engineering techniques such as Raman spectroscopy, SEM with EDS, and XRD. These studies allowed the identification of pyroxenes, olivines, plagioclase, and iron oxides—minerals typical of aubrite.

The most unexpected result of the conducted analyses is the identification in the Tiglit aubrite of calcite, quartz, cristobalite, and carbon in the form of nanodiamonds.

The identification of high-temperature SiO_2_ polymorphs is particularly noteworthy given the highly reducing conditions under which aubrites typically form, where free silica is thermodynamically unstable. The presence of high-temperature SiO_2_ polymorphs suggests a complex post-formational history involving shock metamorphism, transient heating, and rapid cooling, potentially linked to impact events or localized redox heterogeneities. The presence of carbon material (evidenced by the presence of D and G bands in Raman spectra) is further confirmation that the parent body suffered multiple collisions in space along its path.

EDS analysis confirmed the presence of sulfide phases within the Tiglit aubrite, consistent with previous reports in the literature on aubritic mineralogy. However, Raman spectra do not allow for the clear identification of sulfide-related bands due to their overlap with signals from silicates and other phases, highlighting the need for further studies.

## Figures and Tables

**Table 1:** Results of chemical composition analysis for selected areas of the meteorite surface marked in Figure 1 and a fragment of powdered meteorite.

Number	Atomic % of Element													
	C	N	O	Na	Mg	Al	Si	S	Fe	Cu	Ti	Cl	K	Ca
measurements on the surface in area A														
A0	10.80		46.60		12.6	0.80	13.70	7.00	7.90					
A1	18.09	-	21.33	-	0.79	-	1.08	9.09	49.61	-	-	-	-	-
A2	15.69	-	28.70	-	3.23	0.53	2.94	7.18	41.72	-	-	-	-	-
A3	12.90	-	34.72	-	2.75	1.91	5.53	12.39	29.8	-	-	-	-	-
A4	-	-	59.79	7.19	0.45	8.48	24.09	-	-	-	-	-	-	-
A5	11.55	-	61.67	-	14.48	0.41	11.90	-	-	-	-	-	-	-
measurements on the surface in area B														
B0	17.10		44.10		11.5	0.6	11.9	6.8	8.0					
B1	12.63	-	29.36	-	7.08	-	7.04	19.92	23.57	0.41	-	-	-	-
B2	27.02	-	22.90	-	1.46	-	1.91	9.9	36.81	-	-	-	-	-
B3	16.20	-	30.20	-	6.43	0.60	5.65	14.86	26.06	-	-	-	-	-
B4	8.72	-	59.99	-	14.75	0.87	15.28	0.40	-	-	-	-	-	-
B5	-	-	52.55	-	23.33	-	24.12	-	-	-	-	-	-	-
measurements on the surface in area C														
C0	16.06	-	11.07	-	-	-	-	21.04	51.82	-	-	-	-	-
C1	-	-	46.8	-	12.77	0.37	11.93	11.37	16.75	-	-	-	-	-
C2	13.97	-	38.55	-	9.71	-	9.63	10.9	17.24	-	-	-	-	-
C3	-	-	59.31	-	19.79	-	20.90	-	-	-	-	-	-	-
C4	62.20	-	29.87	0.98	1.67	1.09	3.36	0.58	-	-	-	0.25	-	-
C5	35.25	-	31.71	-	4.63	-	4.01	0.40	-	-	24.00	-	-	-
measurements on the surface in area D														
D0	16.28	-	24.61	-	1.27	2.99	5.92	22.58	26.35	-	-	-	-	-
D1	25.61	-	24.36	-	-	-	1.87	14.10	34.05	-	-	-	-	-
D2	15.27	-	50.58	-	15.84	0.90	17.41	-	-	-	-	-	-	-
D3	72.24	-	11.50	0.78	-	-	-	0.88	-	-	13.4	1.21	-	-
D4	59.35	9.37	17.67	-	1.20	-	0.89	0.51	-	-	10.79	0.22	-	-
D5	53.79	6.56	29.45	0.38	3.75	0.44	4.24	0.22	0.47	-	-	0.22	0.26	0.23
powder measurements														
P1	23.66	-	54.33		6.83	-	7.29	0.29	0.13	-	-		-	7.46
P2	18.95	-	55.82	0.84	7.62	-	8.17	0.42	-	-	-	-	-	8.18
P3	23.88	-	54.48		6.81	-	7.07	0.23	0.10	-	-	-	-	7.43
P4	30.37	-	48.53	0.71	6.46	0.47	6.62	-	-	-	-	-		6.85
P5	23.80	-	54.49		6.77	-	7.08	0.26	0.10	0.04	-	-	-	7.42
P6	29.10	-	50.14	0.75	6.24	0.43	6.38	0.24	0.09	0.05	-	-	-	6.57

## Data Availability

The original contributions presented in this study are included in the article. Further inquiries can be directed to the corresponding author.
